# Long-Term Outcomes for Patients with Prostate Cancer Having Intermediate and High-Risk Disease, Treated with Combination External Beam Irradiation and Brachytherapy

**DOI:** 10.1155/2010/471375

**Published:** 2010-08-18

**Authors:** Michael Dattoli, Kent Wallner, Lawrence True, David Bostwick, Jennifer Cash, Richard Sorace

**Affiliations:** ^1^Dattoli Cancer Center & Brachytherapy Research Institute, 2803 Fruitville Rd., Sarasota, FL 34237-5367, USA; ^2^Department of Radiation Oncology and Pathology, University of Washington, Seattle, WA 98195-0001, USA; ^3^Radiation Oncology, Puget Sound Health Care System, Department of Veterans Affairs, Seattle, WA 98108-1597, USA; ^4^Group Health Cooperative, Seattle, WA 98124-1590, USA; ^5^Research Division, Bostwick Laboratories, Richmond, VA 23050-4410, USA

## Abstract

*Background*. Perception remains that brachytherapy-based regimens are inappropriate for patients having increased risk of extracapsular extension (ECE). *Methods*. 321 consecutive intermediate and high-risk disease patients were treated between 1/92 and 2/97 by one author (M. Dattoli) and stratified by NCCN guidelines. 157 had intermediate-risk; 164 had high-risk disease. All were treated using the combination EBRT/brachytherapy ± hormones. Biochemical failure was defined using PSA >0.2 and nadir +2 at last followup. Nonfailing patients followup was median 10.5 years. Both biochemical data and original biopsy slides were independently rereviewed at an outside institution. *Results*. Overall actuarial freedom from biochemical progression at 16 years was 82% (89% intermediate, 74% high-risk) with failure predictors: Gleason score (*P* = .01) and PSA (*P* = .03). Hormonal therapy did not affect failure rates (*P* = .14). *Conclusion*. This study helps to strengthen the rationale for brachytherapy-based regimens as being both durable and desirable treatment options for such patients. Prospective studies are justified to confirm these positive results.

## 1. Introduction

Whereas modern prostate brachytherapy using transrectal ultrasound and sophisticated treatment planning was met with much skepticism when introduced in the 1980s, favorable longer-term outcomes are fueling its widespread adoption [1, 2]. Brachytherapy has since enjoyed growing acceptance for treatment of low risk patients, that is, those with a PSA less than 10 ng/mL, Gleason score 6, and low volume cancer in the biopsy core specimens [3, 4]. However, there is a continued controversy regarding its use for patients at higher risk of extracapsular cancer extension (ECE) [5–7]. It has been well established that patients with higher PSA and Gleason scores are at higher risk of ECE, with the likelihood of ECE being approximately 50% in patients with a PSA over 10 ng/mL or a Gleason score of 7 or higher [8]. It has been assumed that external beam radiation alone should be the basis of treatment for such patients, the logic being that EBRT would provide better coverage of ECE [9]. Some early reports appeared to support the concept of brachytherapy being ill-advised for these patients [10, 11].

While it may seem superficially logical to avoid brachytherapy for patients at higher risk of ECE, studies regarding the radial extent of ECE have cast doubt on such a policy. Most importantly, ECE is typically limited to 3–5 mm, and can be treated with peripherally loaded implants that provide high doses to the periprostatic tissues [12]. Although some early reports showed poor results using brachytherapy in patients having higher risk features, there is a growing number of reports showing favorable results with brachytherapy-based treatment for patients with a high likelihood of ECE, especially when combined with supplemental EBRT [6, 7, 13–16].

One criticism of most brachytherapy outcomes reports has been the lack of long-term followup, with the possibility of prematurely concluding that cancer has been eradicated in high-risk patients. Reports to date have generally been limited to a median followup of 5 years. Accordingly, we have updated our ongoing analysis of external beam radiation (EBRT) plus brachytherapy using Pd-103.

## 2. Materials and Methods

Three hundred twenty-one consecutive patients were treated between January 1992 and February 1997 by the primary author, and M. Dattoli. 

Patients were classified into prognostic risk groups as defined by the National Comprehensive Cancer Network guidelines (intermediate risk: *T*_2*B*_–*T*_2*C*_ or Gleason 7, or PSA 10–20; high risk: T_3A_ or Gleason 8–10, or PSA above 20). 

The original biopsy slides of the 321 patients were retrieved and rereviewed by outside pathologists (L. True or D. Bostwick) to independently verify the patients' tumor grade with 30% identified to be undergraded and 6% being overgraded. All biochemical data were also independently re-reviewed at the University of Washington (K.W.). Only one patient had a staging pelvic lymphadenectomy. Postimplant saturation biopsies were only performed for patients having a rising PSA. Enzymatic prostatic acid phosphatase (PAP) was analyzed independent of risk stratification grouping as the primary author (M. Dattoli) has previously identified the importance of this marker as an adverse prognosticator [6, 17, 18]. PAP was determined by the method of Roy and colleagues, with values up to 2.5 U considered normal [17, 18]. All patients who met intermediate or high-risk criteria underwent combination external beam irradiation plus Pd-103 brachytherapy, with the only exception being that of patients having enlarged gland sizes in excess of 70 cm^3^ and/or excessive TURP defects. Sixty patients had pretreatment TURPS and 30 patients had pretreatment TUIPS.

Patients received a median 4140 cGy 3-dimensional conformal radiotherapy (3D-CRT) over 4.5 weeks to the pelvic field covering the prostate, seminal vesicles, and lymph nodes up to the common iliacs (dose range: 39–60 Gy, 180 cGy/Fx), followed 2 to 4 weeks later by a Pd-103 boost, using transrectal ultrasound and fluoroscopic guidance. Only free seeds were utilized and all patients underwent pretreatment and intraoperative TRUS planning, while all patients underwent postimplant CT imaging for dosimetric analysis and source counting on postoperative Day 1. Extraprostatic seed placement was routinely performed as described by Dattoli et al. [19] The prescribed minimum Pd-103 dose to the prostate was 80–90 Gy (pre-NIST-99). A median of 104.3 mCi Pd-103 was implanted with a range of 48–144 mCi. The median source strength was 1.4 mCi (range:1.0–1.6 mCi/source). Generous brachytherapy margins were utilized; the clinical target volume extended 0.5–1.0 cm anterolaterally to the TRUS prostate margin (no posterior margin was added beyond the TRUS delineated posterior border). Patients having 3 or more risk features (PSA, Gleason score, Clinical Stage, elevated PAP) were encouraged to receive hormonal agents and 143 patients received hormones in neoadjuvant or adjuvant fashion, median duration 4 months (maximum 6 months). Patients were planned to be followed at 6 and 12 months for the first 5 years, and then every 12 months thereafter. At the time of their follow-up visit, data recorded included their IPSS and rectal functioning assessment score (R-FAS). Those patients who did not appear for their scheduled follow-ups (beyond 12 months, 20 patients) were mailed the IPSS and R-FAS. Ultimately, all living patients in this study were evaluated by personal visits with the longest time lapse being 18 months. Eighteen patients experiencing Grade II proctitis beyond 6 months were recommended to undergo colonoscopy to rule out fistulas or ulceration, revealing only erythematous changes and/or prominent internal hemorrhoids.

Freedom from biochemical failure was defined using a serum PSA <  0.2 ng/mL (at or after nadir) at last follow-up. The *Phoenix* definition of PSA nadir +2 was also used. Using PSA < 0.2 ng/mL for disease failure allows for reasonable comparison to radical prostatectomy series, as well as recent brachytherapy studies while PSA nadir +2 allows for comparison to contemporary IMRT series [20–22]. 

Patients were censored at last follow-up if their serum PSA was still decreasing (3 patients). Patients whose PSA nadired > 0.2 ng/mL or who exceeded the PSA nadir +2 definition were scored as failures at the time at which their PSA progressed. Both definitions (PSA <0.2 ng/mL and PSA nadir +2) needed to be satisfied to be considered free from biochemical failure. The follow-up period for nonfailing patients was 16 years (median 10.5 years). Biochemical failure curves were calculated by the method of Kaplan-Meier and freedom from biochemical failure was defined as both a PSA < 0.2, or a rise in PSA that does not exceed the PSA nadir +2 definition. Differences between groups were determined by log-rank method. 

Individual NCCN risk factors (Clinical Stage, Gleason score, PSA) were also subject to multivariate Cox proportional hazards analysis considering each factor as a continuous variable. Treatment with or without hormones was subject to the methods of Kaplan-Meier. Since this primary author (M. Dattoli) has previously identified PAP as a significant adverse prognosticator, this risk feature was also subjected to the same multivariate analysis.

## 3. Results

157 patients had intermediate risk disease and 164 had high risk disease. This included 218 patients having Gleason Score 7 or greater with 52 patients having Gleason 8–10; 203 patients having PSA > 20; 130 patients had clinical stage T_3_; 158 patients had clinical stage T_2c _; 20 patients had clinical stageT_2b_; and 10 patients had clinical stage T_2a_ and 3 patients had clinical stage T_1c_ (Table 1). Seventy-nine patients had abnormally elevated PAP's. Patient ages ranged from 43 to 88 years (median: 66 years).

**Figure 1:**
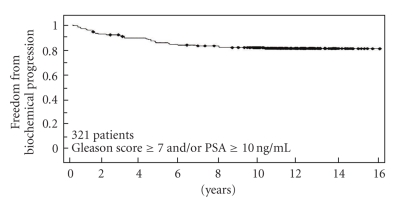
Combined freedom from biochemical progression (using PSA nadir +2, PSA < 0.2 at last follow-up) when evaluated for all 321 patients treated with PD-103 plus median 41 Gy beam radiation. (No significant variance was identified when plotting graphs using the two definitions.) (*univariate analysis*).

The overall actuarial freedom from biochemical progression at 16 years is 82%, with 222 patients followed beyond five years and 149 patients followed beyond 10 years (Figure 1). The overall freedom-from-failure for the 157 patients with intermediate risk disease was 89% while the overall freedom-from-failure for the 164 patients having high risk features was 74% at 16 years. It was most encouraging to find that the absolute risk of failure decreased progressively with time, with only 1% of patients failing beyond 6 years of completing treatment. Fifty-two patients developed biochemical failure. Of these 52 patients, 27 (51%) failed within the first three years after treatment. Follow-up transperineal saturation prostate biopsies (minimum 25 cores) were performed on all failing patients, within 2 months of biochemical failure (MD). There were no pathologically documented local failures. None were characterized as “indeterminate” (DB) nor was there clinical (DRE, new onset of obstructive uropathy, pelvic pain) evidence of local failure.

**Figure 2:**
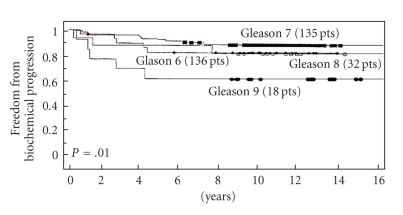
Freedom from biochemical progression (PSA <  0.2 ng/mL, nadir +2) stratified per Gleason score (*multivariate analysis*).

**Figure 3:**
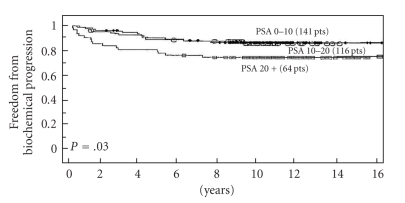
Freedom from biochemical progression (PSA < 0.2 ng/mL, nadir +2) stratified per PSA elevation (*multivariate analysis*).

**Figure 4:**
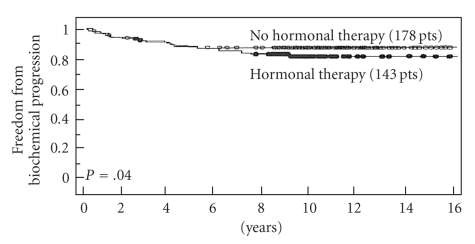
Freedom from biochemical progression (PSA <  0.2 ng/mL, nadir +2) with or without adjuvant hormonal therapy (*univariate analyis*).

Of the 3 NCCN risk features (PSA, Gleason score, and clinical stage) only pretreatment PSA and Gleason score were each associated with a higher failure rate (Table 2). There was no statistical significance between clinical stage (*P* = .4). The strongest predictor of failure was Gleason score (*P* = .03) (Figure 2) and PSA (*P* = .041) (Figure 3). Neoadjuvant and adjunctive hormonal therapy did not affect the failure rates (*P* = .14, Figure 4). Consistent with this author's (M.D.) previous experience, PAP was identified to be the strongest predictor of biochemical failure (*P* = .001).

Postimplant prostatic dosimetric evaluation was performed on all patients demonstrating mean and median V100's to be 99.5 and 98.3, respectively, while mean and median D90's were 105 and 102, respectively.

Treatment morbidity was primarily limited to 3–6 months RTOG grade 1-2 urinary and rectal symptoms (80% RTOG grade 1, 20% grade 2). These symptoms occurred 3–6 months following completion of treatment and all spontaneously resolved with the exception of one patient who developed RTOG grade 3 toxicity. This patient experienced chronic intermittent urinary obstruction symptoms and had both a posttreatment TUIP and TURP resulting in low volume stress incontinence. Fifteen patients required immediate postimplant catheterization limited to 24–48 hours. None required indwelling catheterization beyond 48 hours and none required repeated self-catheterization. No patient developed rectal fistula or ulceration.

## 4. Discussion

The reintroduction of brachytherapy in the late 1980s was met with tremendous skepticism and misconceptions regarding which patients, if any, are best served with this modality. Skepticism has since given way to widespread acceptance of brachytherapy alone for patients with low PSA and Gleason score [2]. However, there still remains widespread perception that brachytherapy is not appropriate for patients at higher risk of ECE. A recent European consensus statement, for instance, recommended that brachytherapy be limited to patients with low PSA and Gleason scores [3]. On the contrary, this and other series suggest that brachytherapy-based treatment may, in fact, be a very desirable treatment modality for such patients when performed in combination with EBRT. Brachytherapy, if designed to deliver generous cancercidal margins around the prostate given in addition to those with supplemental EBRT, appears capable of eradicating both larger intraprostatic tumor masses along with ECE [22, 23]. Hormones offered no survival advantage (*P* = .14) in keeping with multiple other brachytherapy studies using high dose radiation [6, 24–27]. This is in contrast to recent studies demonstrating survival advantage using lower doses as is the case with full course external beam irradiation and androgen blockade without brachytherapy, including the Trans-Tasman randomized control trial (median 6 months hormonal manipulation [28, 29]) and other studies using a median of 2 years and 3 years [30–32].  Because there is a large degree of overlap in PSA, Gleason score and Clinical Stage between patients with or without biochemical failure, even patients with markedly elevated parameters appear to have a chance for cure. Accordingly, our policy is to treat patients with curative intent, even with markedly elevated parameters, provided a bone scan and pelvic CT are negative for metastatic disease. 

Recent data suggest that in those patients having high risk malignancy, the disease is predominantly confined within the pelvis including the prostate and periprostatic tissues, seminal vesicles, and pelvic lymph nodes [6, 33, 34]. Based on results obtained from our study published in 1996 which utilized preimplant external radiation followed by brachytherapy, beginning in February 1997, we began boosting periprostatic tissues and lymph nodes up to the common iliacs to a much higher cancercidal dose level (60–75 Gy) while blocking the prostate plus a calculated margin [35]. This method was applied to the final 7 patients in this study. This dose escalation boost has become more easily accomplished with the advent of the more sophisticated versions of IMRT. 

Initial studies demonstrate favorable tumor control rates with brachytherapy-based regimens were met with skepticism due to short follow-up times. However, the increasing number of studies with long-term follow-up uniformly achieves results that compare favorably to those with surgery or beam radiation alone. It is encouraging that the failure rate decreased to <1% per year (90% of surviving patients having PSA's <  .05 and the remainder having PSA's of <.2) with follow-up beyond 6 years with no pathological local failures documented. While longer follow-up will always be beneficial, evidence from this patient group at higher risk of extracapsular cancer extension and others suggest that relatively high tumor control rates with brachytherapy-based therapy are quite durable [6, 13, 14, 16]. This increasing body of evidence strengthens the rationale that brachytherapy-based treatment is a very desirable treatment modality for patients having intermediate and high risk disease, although prospective studies will ultimately be necessary to corroborate these positive results.

## Figures and Tables

**Table 1:** Disease characteristics for 321 patients.

Characteristics	Patients (*n*)
Clinical Stage:	
T_3_	130
T_2c_	158
T_2b_	20
T_2a_	10
T_1c_	3

Gleason:	
7	218
8	32
9-10	18

PSA:	
0–10	141
10–20	116
>20	64

**Table 2:** Predictors of Biochemical Failure (multivariate analysis).

Gleason Score	0.03
PSA	0.41
T-Stage	0.4
PAP	0.001
